# Modeling and Calibration for Dithering of MDRLG and Time-Delay of Accelerometer in SINS

**DOI:** 10.3390/s22010278

**Published:** 2021-12-30

**Authors:** Jinlong Xing, Gongliu Yang, Tijing Cai

**Affiliations:** 1 School of Instrument Science and Engineering, Southeast University, Nanjing 210018, China; 230198299@seu.edu.cn (J.X.); yanggongliu@buaa.edu.cn (G.Y.); 2 School of Instrumentation and Optoelectronic Engineering, Beihang University, Beijing 100191, China

**Keywords:** size effect, system-level calibration, inertia lnavigation, lasergyro, dither

## Abstract

At present, the design and manufacturing technology of mechanically dithered ring laser gyroscope (MDRLG) have matured, the strapdown inertial navigation systems (SINS) with MDRLG have been widely used in military and business scope. When the MDRLG is working, high-frequency dithering is introduced, which will cause the size effect error of the accelerometer. The accelerometer signal has a time delay relative to the system, which will cause the accelerometer time delay error. In this article, in order to solve the above-mentioned problem: (1) we model the size effect error of the mechanically dithering of the MDRLG and perform an error analysis for the size effect error of the mechanically dithering of the MDRLG; (2) we model the time delay error of accelerometer and perform an error analysis for the time delay error of accelerometer; (3) we derive a continuous linear 43-D SINS error model considering the above-mentioned two error parameters and expand the temperature coefficients of accelerometers, inner lever arm error, outer lever arm error parameters to achieve high-precision calibration of SINS. We use the piecewise linear constant system (PWCS) method during the calibration process to prove that all calibration parameters are observable. Finally, the SINS with MDRLG is used in laboratory conditions to test the validity of the calibration method.

## 1. Introduction

Error parameters of the inertial device are important factors affecting the navigation accuracy of SINS. Any small error parameter of the inertial device will cause a large navigation error through the divergence of the algorithm error, which needs to be compensated by calibration. Error parameter calibration methods mainly include discrete calibration and systematic calibration. The discrete calibration relies on the accurate azimuth, position, and angular rate reference provided by the high-precision turntable, and by referring to the local gravity acceleration and the earth’s rotation angular rate, placing the IMU in different positions can calibrate the error terms of the gyroscopes and accelerometers [1,2]. However, the calibration accuracy of the discrete calibration is fundamentally limited by the accuracy of the turntable [3], and the cost of high-precision turntable equipment is too high, which makes it difficult to greatly improve the accuracy of the discrete calibration.

The systematic calibration method is the process of estimating the SINS error parameters from the navigation error (attitude error, velocity error, position error) of the SINS that is based on the error model of the SINS. Systematic calibration does not rely on high-precision turntables, so it has been widely used in self-calibration and field calibration of SINS. Pittman [4] pointed out the four major advantages of the systematic calibration method: it can realize the on-site calibration of the SINS; it can realize the self-calibration of the SINS; it does not require high-precision turntables and other high-precision test equipment; it does not need to measure and record the output of the gyroscope or accelerometer. At present, the research on systematic calibration mainly focuses on the layout of the calibration path and the design of the Kalman filter. The main purpose of the calibration path is to decouple the error transmission and improve the observability of error parameters. Savage [5] designed 2 sets of rotation sequences to compare the specific force components before and after the rotation, so as to achieve the calibration of IMU error parameters. Zhanghua Zhou [6] proposed a 10-position calibration method, which has low requirements on the accuracy of the turntable. Chamberlain L [7] designed an 18-position calibration scheme, which can attain the calibration accuracy requirements of navigation-level SINS within 20 min and is currently widely used. Joos [8] pointed out that the systematic calibration method can improve the calibration accuracy of the space microgravity measurement accelerometer. Grewal [9] designed a 51-dimensional state filter and a 12-dimensional state filter to estimate the gyroscope parameters and accelerometer parameters. Cai analyzed the influence of the accelerometer’s nonlinear scaling factor in the calibration based on norm-observation and used the particle swarm algorithm to solve the problem of solving complex nonlinear observation equations [10]. Yu Xudong [11] used the latitude and longitude error as the observation to accurately identify the drift of the laser gyroscope. Liu Bing [12] designed a systematic calibration method based on a high-order Kalman filter algorithm. Shi Wenfeng [13] designed a ten-position systematic calibration path and established a 33-dimensional Kalman filter to estimate the error parameters. Yu Hailong [14] designed a 33 dimensional Kalman filter, which considered the quadratic error coefficient of the accelerometer, and verified it through simulation experiments.

Due to the mechanical structure and installation error, the sensitive points of the three accelerometers and the sensitive point of IMU do not coincide. So the measuring points of the three accelerometers are different, which will cause navigation errors. This error effect is called the inner lever arm effect. Weng Jun included the non-synchronization of the accelerometer in Kalman’s state variables and perfected the calibration error model [15]. Literature [16,17,18] considered the size effect of the accelerometer and the accelerometer-gyro time asynchronous error. Literature [19] designed a 25-position rotation arrangement method, which additionally considered the quadratic error of the accelerometer and the error of the inner lever arm, and verified the feasibility of the calibration path. The above-mentioned internal lever arm effect research is aimed at the fixed lever arm compensation under the static state of the gyroscope. When the MDRLG working, high-frequency dithering is introduced, which will cause the size effect error of the accelerometer. At present, the research on the compensation method of the size effect caused by the dithering of the MDRLG is rare.

Since the signal of the accelerometer needs to pass a Voltage Frequency Converter (VF) or a Current Frequency Converter (IF), there is a time delay relative to the gyroscope signal. The asynchronous time between gyroscope and accelerometer would generate navigation errors. Literature [20,21] modeled asynchronous time between auxiliary sensors (such as GPS) and SINS and gave the compensation method. At present most studies about the compensation for the asynchronous time were about the compensation between auxiliary sensors and SINS. However, few studies were about the compensation method of IMU asynchronous time.

In the current study, the error parameters of the accelerometer are considered to be fixed values. Since the accelerometer output is sensitive to the working temperature, the error parameters of the accelerometer will change accordingly when the accelerometer is working in an environment with drastic temperature changes. so it is necessary to compensate the error parameters of the accelerometer to eliminate the calibration parameter errors caused by temperature changes. The currently widely used temperature compensation method mainly uses polynomial fitting to obtain the fitting relationship between the output of the accelerometer and the temperature, and then establishes the temperature error model of the zero offsets and the scale factor [22] and compensates in the algorithm. Research on temperature error compensation in calibration is still rare.

In order to solve the above-mentioned problems, in this article, we analyze the impact of the dithering of laser MDRLG on navigation accuracy. The analysis results show that during the dithering along the MDRLG sensitive axis, the mechanical dithering will cause the size effect error of the accelerometers on the other two axes and the solution is given. We model the size effect error of the accelerometer caused by the mechanical dithering of MDRLG and gave the error compensation method. Since there is a slight time delay between the signal of the MDRLG and the signal of the accelerometer, which will cause navigation time delay error. In this article, we analyze the impact of the signal’s time delay of the accelerometer on navigation error and give a calibration method for the time delay. Since the output of the accelerometer is sensitive to the working temperature, when the accelerometer is working in a temperature-changing environment, the error parameters of the accelerometer are changing. An error model including temperature coefficient is established to solve the problem that the error parameters of accelerometer vary with temperature. Finally, on the basis of the above-mentioned errors, we also consider the inner and outer lever arm errors and derive the continuous linear SINS error model considering the above-mentioned error parameters, and use the Kalman filter to estimate the error parameters. A 43 dimensional (43-D) filter is designed to realize the accurate estimation of the above-mentioned error, and the observability of the proposed state is analyzed using PWCS and Singular Value Decomposition (SVD) methods.

## 2. Reference Frame Definition

The reference coordinate frames involved in this article are defined as follows.

*i* Coordinate frame: Earth-centered initially fixed (ECIF) orthogonal reference coordinate.

*e* Coordinate frame: Earth-centered earth fixed (ECEF) orthogonal reference coordinate.

*b* Coordinate frame: Orthogonal reference coordinate aligned with right– forth–up (RFU) axes.

*n* Coordinate frame: Orthogonal reference coordinate aligned with actual east–north–up (ENU) geodetic axes.

*g* Coordinate frame: Nonorthogonal reference coordinate aligned with gyro-sensitive axes.

*a* Coordinate frame: Nonorthogonal reference coordinate aligned with accelerometer-sensitive axes.

## 3. Modeling and Error Analysis for Systematic Calibration of Sins

### 3.1. System Configuration of the SINS

**Figure 1:**
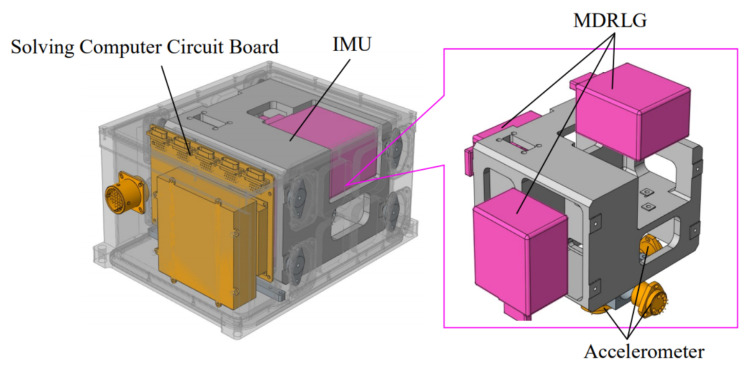
SINS with MDRLG structure.

The system configuration for SINS with MDRLG is shown in Figure 1. The SINS mainly includes a solving computer circuit board and an Inertial Measurement Unit (IMU). The IMU consists of three MDRLGs and three accelerometers. The three MDRLGs and accelerometers are placed in an orthogonal structure. Due to the error in the machining of the orthogonal structure. The MDRLGs and accelerometers installations are nonorthogonal. The unit vectors of the sensitive axes of three MDRLGs are $x^{b}$, $y^{b}$ and $z^{b}$, respectively. The unit vectors of the sensitive axes of three accelerometers are $x^{a}$, $y^{a}$ and $z^{a}$, respectively.

### 3.2. Modeling and Error Analysis for Dithering of the MDRLG

**Figure 2:**
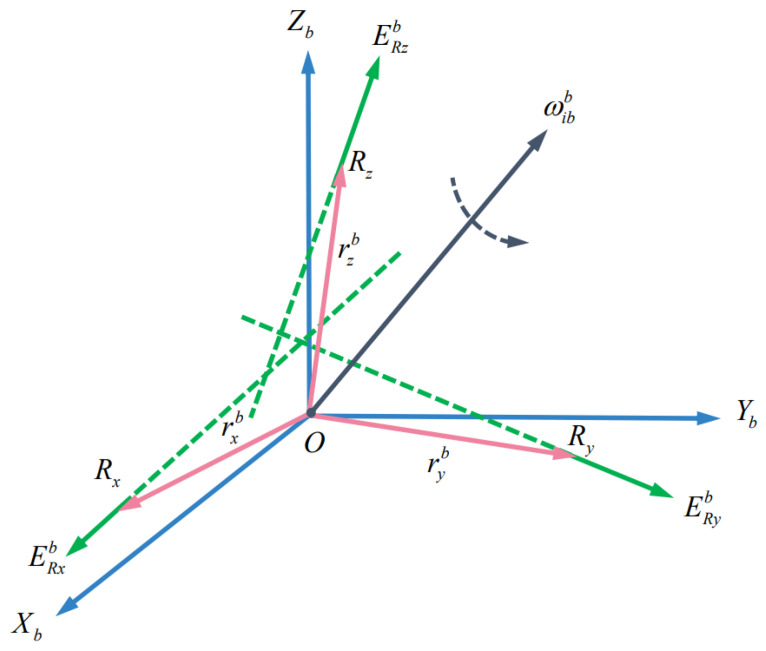
Schematic diagram of size effectt.

It is assumed that the three accelerometers of the SINS are installed in the *b*($OX_{b}Y_{b}Z_{b}$) coordinate frame. As shown in Figure 2, the sensitive points of the three accelerometers are $R_{x}$, $R_{y}$, and $R_{z}$. The position vectors relative to the sensitive point *O* of the system(origin of the *b*($OX_{b}Y_{b}Z_{b}$) coordinate frame) are $r_{x}^{b}$, $r_{y}^{b}$ and $r_{z}^{b}$, and the unit vectors in the direction of the accelerometer’s sensitive axes are $E_{Rx}^{b}$, $E_{Ry}^{b}$, and $E_{Rz}^{b}$. When the rotational angular velocity of the system relative to the inertial space is $\omega_{ib}^{b}$, the output of the three accelerometers is as follows:

(1) $$f_{RI}^{b}=\left[f_{o}^{b}+\omega_{ib}^{b}\times \left(\omega_{ib}^{b}\times r_{I}^{b}\right)+{\dot{\omega }}_{ib}^{b}\times r_{I}^{b}\right]\cdot E_{R}^{b}\left(I=x,y,z\right)$$

In Equation (1), $f_{RI}^{b}$ represents the output of the *I*-axis accelerometer in the *b* coordinate frame, and $f_{o}^{b}$ represents the acceleration at point *O* in the *b* coordinate frame.

Hypothesize $f_{oI}^{b}=f_{o}^{b}\times E_{RI}^{b}$. Substituting Equation (2) into Equation (1), Equation (1) can be written as follows:

(2) $$f_{rI}^{b}=f_{RI}^{b}-f_{ol}^{b}=\left[\omega_{ib}^{b}\times \left(\omega_{ib}^{b}\times r_{I}^{b}\right)\right]\times E_{RI}^{b}+\left[{\dot{\omega }}_{ib}^{b}\times r_{I}^{b}\right]\times E_{RI}^{b}$$

Equation (2) is the expression of the accelerometer size effect error, where the first term on the right is the centripetal acceleration, and the second term is the tangential acceleration. It can be seen that the size effect error is the additional interference acceleration generated by the accelerometer installation deviation $r_{I}^{b}$ under the action of the angular movement of the SINS. This error is proportional to the distance of the accelerometer from the sensitive point of the system.

After the installation error of the inertial device of the SINS is compensated, the sensitive axes of three accelerometers and three MDRLGs are, respectively, parallel and consistent with the $OX_{b}$-axis, $OY_{b}$-axis and $OZ_{b}$-axis of the *b* coordinate frame, then

(3) $$\left\{\begin{array}{c} E_{Rx}^{b}=\left[\begin{array}{ccc} 1 & 0 & 0 \end{array}\right]\\ E_{Ry}^{b}=\left[\begin{array}{ccc} 0 & 1 & 0 \end{array}\right]\\ E_{Rz}^{b}=\left[\begin{array}{ccc} 0 & 0 & 1 \end{array}\right] \end{array}\right.$$

Hypothesize

(4) $$\begin{array}{c} \omega_{ib}^{b}={\left[\begin{array}{ccc} \omega_{ibx}^{b} & \omega_{iby}^{b} & \omega_{ibz}^{b} \end{array}\right]}^{T},\\ r_{x}^{b}={\left[\begin{array}{ccc} r_{xx}^{b} & r_{xy}^{b} & r_{xz}^{b} \end{array}\right]}^{T},\\ r_{y}^{b}={\left[\begin{array}{ccc} r_{yx}^{b} & r_{yy}^{b} & r_{yz}^{b} \end{array}\right]}^{T},\\ r_{z}^{b}={\left[\begin{array}{ccc} r_{zx}^{b} & r_{zy}^{b} & r_{zz}^{b} \end{array}\right]}^{T} \end{array}$$

By substituting Equations (3) and (4) into Equation (2), we can obtain the size effect error of acceleration in the b coordinate frame as follows:

(5) $$f_{r}^{b}=\left[\begin{array}{c} f_{rx}^{b}\\ f_{ry}^{b}\\ f_{r=}^{b} \end{array}\right]=\left[\begin{array}{c} -r_{xx}^{b}\left[{\left(\omega_{\mathrm{iby}}^{b}\right)}^{2}+{\left(\omega_{\mathrm{ibz}}^{b}\right)}^{2}\right]+r_{xy}^{b}\left(\omega_{\mathrm{ibx}}^{b}\omega_{\mathrm{iby}}^{b}-{\dot{\omega }}_{\mathrm{ibz}}^{b}\right)+r_{xz}^{b}\left(\omega_{\mathrm{ibx}}^{b}\omega_{\mathrm{ibz}}^{b}+{\dot{\omega }}_{\mathrm{iby}}^{b}\right)\\ r_{yx}^{b}\left(\omega_{\mathrm{ibx}}^{b}\omega_{\mathrm{iby}}^{b}+{\dot{\omega }}_{\mathrm{ibz}}^{b}\right)-r_{yy}^{b}\left[{\left(\omega_{\mathrm{ibx}}^{b}\right)}^{2}+{\left(\omega_{\mathrm{ibz}}^{b}\right)}^{2}\right]+r_{yz}^{b}\left(\omega_{\mathrm{iby}}^{b}\omega_{\mathrm{ibz}}^{b}-{\dot{\omega }}_{\mathrm{ibx}}^{b}\right)\\ r_{ix}^{b}\left(\omega_{\mathrm{ibx}}^{b}\omega_{\mathrm{ibz}}^{b}-{\dot{\omega }}_{\mathrm{iby}}^{b}\right)+r_{zy}^{b}\left(\omega_{\mathrm{iby}}^{b}\omega_{\mathrm{ibz}}^{b}+{\dot{\omega }}_{\mathrm{ibx}}^{b}\right)-r_{zz}^{b}\left[{\left(\omega_{\mathrm{ibx}}^{b}\right)}^{2}+{\left(\omega_{\mathrm{iby}}^{b}\right)}^{2}\right] \end{array}\right]$$

The frequency offset technology of mechanical dithering is usually used to overcome the lock-up effect of the MDRLG and the SINS with MDRLG is always in a high-frequency dynamic motion state, which will cause the size effect error of the accelerometer. The signal of MDRLG is processed by an internal digital filtering algorithm and the output of MDRLG only contains the actual angular velocity $\omega_{ib}^{b}$ of the SINS. Therefore, due to the lack of measurement information of the angular velocity of the dithering of MDRLG, it is difficult to algorithmically compensate for the size effect error of the accelerometer caused by the dithering of MDRLG. The corresponding research and solution can only be carried out in the IMU structure design, error compensation, and calibration test. The dithering signal of the MDRLG can generally be expressed as:

(6) $$\omega_{ib}^{b}=A\cos\left(\Omega t\right)$$

In Equation (6), $\omega_{ib}^{b}$ is the angular velocity of the dithering of MDRLG; *A* is the amplitude of the dithering of MDRLG; Ω is the frequency of the dithering of MDRLG; *t* is the time.

For the convenience of analysis, only the size effect error of the accelerometer caused by MDRLG on one axis is considered. Suppose the angular velocity caused by $o_{b}x_{b}$-axis MDRLG is $\omega_{jx}$, the amplitude of the dithering of $o_{b}x_{b}$-axis MDRLG is $A_{x}$, the frequency of the dithering of $o_{b}x_{b}$-axis MDRLG is $\Omega_{x}$, and the angular velocity of the dithering of MDRLG on the other two axes is 0, then

(7) $$\omega_{ib}^{b}=\left[\begin{array}{c} \omega_{jx}\\ 0\\ 0 \end{array}\right]=\left[\begin{array}{c} A_{x}\cos\left(\Omega_{x}t\right)\\ 0\\ 0 \end{array}\right]$$

By substituting Equation (7) into Equation (5), the size effect error of the accelerometer caused by the dithering of $o_{b}x_{b}$-axis MDRLG can be obtained as:

(8) $$\delta f_{rs}^{b}=\left[\begin{array}{c} \delta f_{rsx}^{b}\\ \delta f_{rsy}^{b}\\ \delta f_{rs=}^{b} \end{array}\right]=\left[\begin{array}{c} 0\\ \frac{-r_{yy}^{b}A_{x}^{2}}{2}+r_{yz}^{b}A_{x}\Omega \sin\left(\Omega t\right)+\frac{-r_{yy}^{b}A_{x}^{2}}{2}\cos\left(2\Omega t\right)\\ \frac{-r_{zz}^{b}A_{x}^{2}}{2}-r_{zy}^{b}A_{x}\Omega \sin\left(\Omega t\right)+\frac{-r_{zz}^{b}A_{x}^{2}}{2}\cos\left(2\Omega t\right) \end{array}\right]$$

It can be seen from Equation (8) that the dithering of $o_{b}x_{b}$-axis MDRLG will cause the size effect errors of the accelerometers on $o_{b}y_{b}$-axis and $o_{b}z_{b}$-axis. The size effect errors are in the form of oscillation, consisting of a constant value component, a first-order component of the dithering frequency, and a second-order component of the dithering frequency. The main factor affecting the system accuracy is the constant component, which is mainly related to the dithering amplitude of MDRLG, and the relationship with the dithering frequency of MDRLG is not obvious. We can write the velocity and position errors as follows:

(9) $$\delta v^{ns}=\left[\begin{array}{c} 0\\ \frac{-r_{yy}^{b}A_{x}^{2}}{2}t\\ \frac{-r_{zz}^{b}A_{x}^{2}}{2}t \end{array}\right]\;\;\delta P^{ns}=\left[\begin{array}{c} 0\\ \frac{-r_{yy}^{b}A_{x}^{2}}{2}t^{2}\\ \frac{-r_{zz}^{b}A_{x}^{2}}{2}t^{2} \end{array}\right]$$

It can be seen from Equation (9) that the velocity error and position error caused by the dithering of the MDRLG are a linear function and a quadratic function of time, respectively.

Assuming that the size effect errors caused by the dithering of the three MDRLG installed on the IMU are the superposition of the size effect error caused by the dithering of each MDRLG alone, the maximum size effect error of the accelerometer caused by the dithering of the three MDRLGs installed on the IMU is

(10) $$\delta f_{r}^{b}=\left[\begin{array}{c} \delta f_{rx}^{b}\\ \delta f_{ry}^{b}\\ \delta f_{r=}^{b} \end{array}\right]=\left[\begin{array}{c} -\frac{r_{xx}^{b}A_{y}^{2}}{2}-\frac{r_{xx}^{b}A_{z}^{2}}{2}\\ -\frac{r_{yy}^{b}A_{x}^{2}}{2}-\frac{r_{yy}^{b}A_{z}^{2}}{2}\\ -\frac{r_{zz}^{b}A_{x}^{2}}{2}-\frac{r_{zz}^{b}A_{y}^{2}}{2} \end{array}\right]=\left[\begin{array}{c} -\frac{\left(A_{y}^{2}+A_{z}^{2}\right)}{2}r_{xx}^{b}\\ -\frac{\left(A_{x}^{2}+A_{z}^{2}\right)}{2}r_{yy}^{b}\\ -\frac{\left(A_{x}^{2}+A_{y}^{2}\right)}{2}r_{zz}^{b} \end{array}\right]$$

In Equation (10), the amplitude $A_{I}\left(I=x,y,z\right)$ can generally be detected and output by the built-in sensor of the MDRLG. It can be seen that the size effect error caused by the dithering of the MDRLG in the IMU is related to the dithering amplitude of the MDRLG installed on the sensitive axis of IMU. When the amplitude of the MDRLGs installed on the three axes of the IMU remains unchanged, the specific force error is a constant value, which is equivalent to the zero-bias of the accelerometer.

The traditional systematic calibration method does not consider the influence of the dithering of MDRLG. The marked zero-bias $\nabla_{I}^{\prime }$ is the combination of the size effect error $\delta f_{rI}^{b}$ caused by the dithering of MDRLG and the real zero-bias $\nabla_{I}$ of the accelerometer, not the real zero offset. The traditional systematic calibration method is performed under the assumption that the amplitude of the dithering of MDRLG is constant. However, in practice, it has been found that the dithering amplitude of MDRLG is affected by vibration, structural environment, and the performance of the dithering mechanism built into the MDRLG. The dithering amplitude of the MDRLG is variable, so it is inaccurate to compensate for the navigation error with constant zero bias. In this article, an error model of the accelerometer considering the size effect error of the dithering of the MDRLG is established, which can calibrate the real zero-bias of the accelerometer. We can see the detailed error model of the accelerometer in Section 3.4.2 and Section 3.4.3. During navigation error compensation, the real zero-bias of accelerometer $\nabla_{I}$ is directly compensated, and the size effect $\delta f_{rI}^{b}$ that caused by the dithering of the MDRLG is calculated and compensated in real-time.

The velocity and position errors caused by the dithering of the MDRLG is shown as follows:

(11) $$\delta v^{n}=\left[\begin{array}{c} (-\frac{r_{xx}^{b}A_{y}^{2}}{2}-\frac{r_{xx}^{b}A_{z}^{2}}{2})t\\ (-\frac{r_{yy}^{b}A_{x}^{2}}{2}-\frac{r_{yy}^{b}A_{z}^{2}}{2})t\\ (-\frac{r_{zz}^{b}A_{x}^{2}}{2}-\frac{r_{zz}^{b}A_{y}^{2}}{2})t \end{array}\right]\;\delta P^{n}=\left[\begin{array}{c} \left(-\frac{r_{xx}^{b}A_{y}^{2}}{2}-\frac{r_{xx}^{b}A_{z}^{2}}{2}\right)t^{2}\\ \left(-\frac{r_{yy}^{b}A_{x}^{2}}{2}-\frac{r_{yy}^{b}A_{z}^{2}}{2}\right)t^{2}\\ \left(-\frac{r_{zz}^{b}A_{x}^{2}}{2}-\frac{r_{zz}^{b}A_{y}^{2}}{2}\right)t^{2} \end{array}\right]$$

It can be seen from Equation (11) that the velocity error and position error caused by the dithering of the MDRLG are a linear function and a quadratic function of time, respectively.

### 3.3. Modeling and Error Analysis for Time Delay of Accelerometer

#### 3.3.1. Error Modeling

For general SINS, it can be considered that the output time of the MDRLG is the sampling time of the system, and the accelerometer signal needs to be converted by IF or VF, so there is a time delay $\delta t_{a}$ between the accelerometer and the system sampling time. Navigation error will be caused by sampling delay of accelerometer when SINS working in a dynamic environment.

The sensitive axes of the accelerometer components are used as the reference of the coordinate frame for specific force updates. The body coordinate frame *b* determined by the MDRLG components is converted to the *B* coordinate frame determined by the sensitive axes of the accelerometers. The theoretical specific force conversion Equation is $f_{SF}^{n}=C_{B}^{n}f_{SF}^{B}$, and the actual calculated value considering the effect of the time delay of the accelerometers is

(12) $$\Delta f_{SF}^{A}=C_{b}^{n}f_{SF}^{A}=C_{b}^{n}C_{B}^{b}f_{SF}^{B}\approx C_{b}^{n}\left[I+\left(\Delta f\right)\times \right]f_{SF}^{B}$$

where

(13) $$\Delta f=\left(\omega_{ib}^{b}-C_{n}^{b}\omega_{in}^{n}\right)\times \delta t_{a}$$

(14) $$\omega_{in}^{n}=\left[\begin{array}{c} -\frac{v_{N}}{R_{M}+h}\\ \omega_{ie}\cos L+\frac{v_{E}}{R_{N}+h}\\ \omega_{ie}\sin L+\frac{v_{N}}{R_{N}+h} \end{array}\right]$$

During the calibration process, the velocity of the IMU is close to zero, and the angular velocity of the earth’s rotation is much smaller than the angular velocity of the turntable. Therefore, we can simplify Equation (13) as

(15) $$\Delta f=\omega_{ib}^{b}\times \delta t_{a}$$

Substitute Equation (15) into Equation (12)

(16) $$\Delta f_{SF}^{n}=C_{b}^{n}\omega_{ib}^{b}\times f_{SF}^{A}\times \delta t_{a}$$

#### 3.3.2. Analysis of Time Delay Error of Accelerometer

Assume that the *b* coordinate frame coincides at the *n* coordinate frame of the initial position of the SINS. The SINS rotates at an angle of ω at a constant speed around the $o_{b}x_{b}$ axis from the initial position. Suppose the posture matrix $C_{b}^{n}$ at time *t* during the rotation is:

(17) $$C_{b}^{n}=\left[\begin{array}{ccc} 1 & 0 & 0\\ 0 & \cos\omega t & \sin\omega t\\ 0 & -\sin\omega t & \cos\omega t \end{array}\right]$$

(18) $$f_{SF}^{A}=\left[\begin{array}{ccc} 0 & g\sin\omega t & -g\cos\omega t \end{array}\right]$$

Ignoring the influence of the earth’s rotation angular velocity $\omega_{ib}$ during the rotation, then $\omega_{ib}={\left[\begin{array}{ccc} \theta_{x} & 0 & 0 \end{array}\right]}^{T}$. Due to mechanical error, the installation of gyro is not orthogonal. Assuming the installation error matrix is $C_{b}^{b2}$, the specific force error generated by the rotation of the SINS is

(19) $$\Delta f_{SF}^{n}=\left[\begin{array}{ccc} 1 & 0 & 0\\ 0 & \cos\omega t & \sin\omega t\\ 0 & -\sin\omega t & \cos\omega t \end{array}\right]\times C_{b}^{b2}\omega_{ib}^{b}\times f_{SF}^{A}\times \delta t_{a}$$

Equation (19) can be simplified as follows:

(20) $$\Delta f_{SF}^{n}=C_{b}^{b2}\delta t_{a}\left[\begin{array}{ccc} 1 & 0 & 0\\ 0 & \cos\omega t & \sin\omega t\\ 0 & -\sin\omega t & \cos\omega t \end{array}\right]\left[\begin{array}{c} \omega \\ 0\\ 0 \end{array}\right]\times \left[\begin{array}{c} 0\\ g\sin\omega t\\ -g\cos\omega t \end{array}\right]=C_{b}^{b2}\delta t_{a}\omega g\left[\begin{array}{c} 0\\ 1\\ 0 \end{array}\right]$$

The velocity and position errors caused by the time delay of accelerometerare can be written as

(21) $$\delta v^{n}=C_{b}^{b2}\delta t_{a}\omega g\left[\begin{array}{c} 0\\ t\\ 0 \end{array}\right]\;\;\delta P^{n}=C_{b}^{b2}\delta t_{a}\omega g\left[\begin{array}{c} 0\\ 0.5t^{2}\\ 0 \end{array}\right]$$

**Figure 3:**
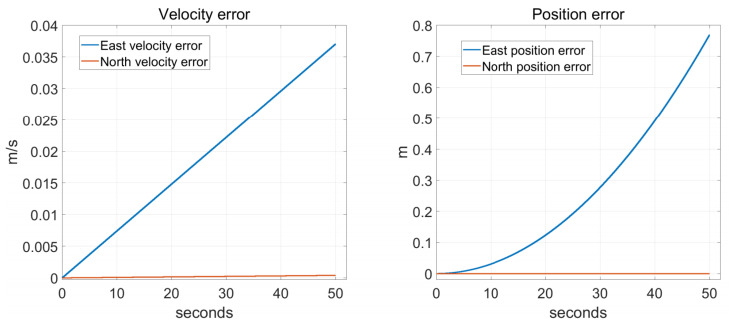
Simulate navigation errors.

It can be seen from the Equation (21) that in the process of rotation, the MDRLG-accelerometer asynchronous time mainly affects the north direction error. In order to verify the correctness of the above derivation, we conduct the following rotation simulation experiment. Assuming that the asynchronous time is 1 ms, the angular velocity of the sensitive axis of each body coordinate frame is $5^{\circ }$/s, the error resolution is 1 × 10${}^{-5}$, and each simulation time is 50 s, the rotation simulation results are shown in Figure 3.

The navigation error propagation obtained by computer simulation in Figure 3 is the same as (21); therefore, the rotation along pitch axes would cause the north navigation error. The above derivation is a relatively ideal situation.

### 3.4. IMU Calibration Parameters and Model of Inertial Device Output Error

#### 3.4.1. Model of IMU Calibration Parameters

The three coordinate axes of the *b* frame (body coordinate frame) are, respectively, $x^{b}$, $y^{b}$ and $z^{b}$, the unit vectors of the three sensitive axes of gyros in the IMU are $x^{g}$, $y^{g}$ and $z^{g}$, respectively. Then the pulse that the MDRLG output per unit time can be written as

(22) $$\left[\begin{array}{c} N_{x}^{g}\\ N_{y}^{g}\\ N_{z}^{g} \end{array}\right]=\left[\begin{array}{ccc} S_{x}^{g} & 0 & 0\\ 0 & S_{y}^{g} & 0\\ 0 & 0 & S_{z}^{g} \end{array}\right]\left[\begin{array}{ccc} x^{g}\cdot x^{b} & x^{g}\cdot y^{b} & x^{g}\cdot z^{b}\\ y^{g}\cdot x^{b} & y^{g}\cdot y^{b} & y^{g}\cdot z^{b}\\ z^{g}\cdot x^{\bar{b}} & z^{}\cdot y^{b} & z^{g}\cdot z^{b} \end{array}\right]\left[\begin{array}{c} \omega_{x}^{b}\\ \omega_{y}^{b}\\ \omega_{z}^{b} \end{array}\right]+\left[\begin{array}{c} b_{x}^{g}\\ b_{y}^{g}\\ b_{z}^{g} \end{array}\right]+\left[\begin{array}{c} n_{x}^{g}\\ n_{y}^{g}\\ n_{z}^{g} \end{array}\right]$$

In Equation (22), $\omega_{ib}^{b}={\left[\begin{array}{ccc} \omega_{x}^{b} & \omega_{y}^{b} & \omega_{z}^{b} \end{array}\right]}^{T}$ is the projection of the input angular velocity vector in the *b* frame, $N^{g}={\left[\begin{array}{ccc} N_{x}^{g} & N_{y}^{g} & N_{z}^{g} \end{array}\right]}^{T}$ is the pulse output per unit time of MDRLG, $S_{I}^{g}$, $b_{I}^{g}$(I=x,y,z) represents the scale factor and zero bias of the *I*-axis MDRLG, respectively, and $M_{g}^{b}=\left[\begin{array}{ccc} x^{g}•x^{b} & x^{g}•y^{b} & x^{g}•z^{b}\\ y^{g}•x^{b} & y^{g}•y^{b} & y^{g}•z^{b}\\ z^{g}•x^{b} & z^{g}•y^{b} & z^{g}•z^{b} \end{array}\right]$ is the dot product of the sensitive axis vector of the MDRLGs and axis vector of the body coordinate frame. The matrix realizes the conversion of the vector from the body coordinate frame to the sensitive-axis coordinate frame of the MDRLGs and reflects the installation relationship of the MDRLGs.

We can set the unit vectors of the three accelerometers sensitive axes as $x^{a}$, $y^{a}$,$z^{a}$ and the pulse that the accelerometer output per unit time can be written as

(23) $$\left[\begin{array}{c} N_{x}^{a}\\ N_{y}^{a}\\ N_{z}^{a} \end{array}\right]=\left[\begin{array}{ccc} S_{x}^{a} & 0 & 0\\ 0 & S_{y}^{a} & 0\\ 0 & 0 & S_{z}^{a} \end{array}\right]\left[\begin{array}{ccc} x^{a}\cdot x^{b} & x^{a}\cdot y^{b} & x^{a}\cdot z^{b}\\ y^{a}\cdot x^{b} & y^{a}\cdot y^{b} & y^{a}\cdot z^{b}\\ z^{a}\cdot x^{b} & z^{a}\cdot y^{b} & z^{a}\cdot z^{b} \end{array}\right]\left[\begin{array}{c} f_{x}^{b}\\ f_{y}^{b}\\ f_{z}^{b} \end{array}\right]+\left[\begin{array}{c} b_{x}^{a}\\ b_{y}^{a}\\ b_{z}^{a} \end{array}\right]+\left[\begin{array}{c} f_{rxm}^{b}\\ f_{rym}^{b}\\ f_{rzm}^{b} \end{array}\right]+\left[\begin{array}{c} n_{x}^{a}\\ n_{y}^{a}\\ n_{z}^{a} \end{array}\right]$$

In Equation (23), $f^{b}={\left[\begin{array}{ccc} f_{x}^{b} & f_{y}^{b} & f_{z}^{b} \end{array}\right]}^{T}$ is the representation of the specific force vector in the *b* frame, $N^{a}={\left[\begin{array}{ccc} N_{x}^{a} & N_{y}^{a} & N_{z}^{a} \end{array}\right]}^{T}$ is the pulse that the accelerometer output per unit time, $S_{I}^{a}$ and $b_{I}^{a}$ are the scale factor and zero bias of the *I*-axis accelerometer, respectively. From Equation (10), it can be seen that $f_{rm}^{b}=\left[\begin{array}{c} f_{rxm}^{b}\\ f_{rym}^{b}\\ f_{r=m}^{b} \end{array}\right]=\left[\begin{array}{c} -0.5r_{xx}^{b}\left(A_{y}^{2}+A_{z}^{2}\right)\\ -0.5r_{yy}^{b}\left(A_{x}^{2}+A_{z}^{2}\right)\\ -0.5r_{zz}^{b}\left(A_{x}^{2}+A_{y}^{2}\right) \end{array}\right]$ is the maximum size effect error of the accelerometer caused by the dithering of MDRLG, $M_{a}^{b}=\left[\begin{array}{ccc} x^{a}•x^{b} & x^{a}•y^{b} & x^{a}•z^{b}\\ y^{a}•x^{b} & y^{a}•y^{b} & y^{a}•z^{b}\\ z^{a}•x^{b} & z^{a}•y^{b} & z^{a}•z^{b} \end{array}\right]$ is the installation relationship matrix of accelerometers, $n_{I}^{a}$ refers to the noise measured by the *I*-axis accelerometer.

Under ideal installation conditions, the sensitive axes of the MDRLGs and the sensitive axes of accelerometers coincide with the axes of the body coordinate frame, respectively. Therefore, the installation relationship matrix $M_{g}^{b}$ and $M_{a}^{b}$ are unit arrays. However, there must be an installation error when the IMU is assembled. Assuming that the installation error angle is a small angle, the installation relationship matrix can be approximately written as:

(24) $$M_{g}^{b}=\left[\begin{array}{ccc} x^{g}•x^{b} & x^{g}•y^{b} & x^{g}•z^{b}\\ y^{g}•x^{b} & y^{g}•y^{b} & y^{g}•z^{b}\\ z^{g}•x^{b} & z^{g}•y^{b} & z^{g}•z^{b} \end{array}\right]\approx \left[\begin{array}{ccc} 1 & -\gamma_{xz}^{g} & \gamma_{xy}^{g}\\ -\gamma_{yz}^{g} & 1 & -\gamma_{yx}^{g}\\ -\gamma_{zy}^{g} & -\gamma_{zx}^{g} & 1 \end{array}\right]$$

(25) $$M_{a}^{g}=\left[\begin{array}{ccc} x^{a}•x^{b} & x^{a}•y^{b} & x^{a}•z^{b}\\ y^{a}•x^{b} & y^{a}•y^{b} & y^{a}•z^{b}\\ z^{a}•x^{b} & z^{a}•y^{b} & z^{a}•z^{b} \end{array}\right]\approx \left[\begin{array}{ccc} 1 & -\gamma_{xz}^{a} & \gamma_{xy}^{a}\\ \gamma_{yz}^{a} & 1 & \gamma_{yx}^{a}\\ -\gamma_{zy}^{a} & \gamma_{zx}^{a} & 1 \end{array}\right]$$

In Equations (24) and (25), $\gamma_{ij}^{g}\left(i,j=x,y,z\right)$ and $\gamma_{ij}^{a}\left(i,j=x,y,z\right)$ are the installation error angles of the MDRLGs and accelerometers, respectively.

The coordinate frame of the turntable has always been used as the reference coordinate frame during discrete calibration. Since the coordinate frame of the turntable is not used as the reference for systematic calibration, a new reference frame must be established and constrained. Take the body coordinate frame $x^{b}y^{b}z^{b}$ as the reference coordinate frame, $x^{b}$ coincides with the sensitive axis unit vector of the MDRLG, $x^{g}$, $y^{b}$ is located in the $x_{g}y_{g}$ plane, $z_{b}$, $x_{b}$ and $y_{b}$ form a right-handed rectangular coordinate frame, the sensitive axis $x^{a}y^{a}z^{a}$ of accelerometers can be projected to the body coordinate frame through 6 angles superior.

Installation errors are considered small angles. Therefore, the relationship between the three sensitive axes of the MDRLGs and the axis of *b* frame can be written as:

(26) $$\begin{array}{c} x^{b}=x^{g}\\ y^{b}=y^{g}+x^{g}\cdot \gamma_{yz}^{g}\\ z^{b}=z^{g}+y^{g}\cdot \gamma_{zx}^{g}-x^{g}\cdot \gamma_{zy}^{g} \end{array}$$

The relationship between the unit vectors of the three sensitive axes of accelerometers can be written as:

(27) $$\begin{array}{c} x^{b}=x^{a}-y^{a}\cdot \gamma_{zx}^{a}+z^{a}\cdot \gamma_{xy}^{a}\\ y^{b}=y^{a}+x^{a}\cdot \gamma_{yz}^{a}-z^{a}\cdot \gamma_{yx}^{a}\\ z^{b}=z^{a}-x^{a}\cdot \gamma_{zy}^{a}+y^{a}\cdot \gamma_{zx}^{a} \end{array}$$

Write Equations (26) and (27) in matrix form as follows:

(28) $$M_{g}^{b}=\left[\begin{array}{ccc} 1 & 0 & 0\\ \gamma_{yz}^{g} & 1 & 0\\ -\gamma_{zy}^{g} & \gamma_{zx}^{g} & 1 \end{array}\right]M_{a}^{b}=\left[\begin{array}{ccc} 1 & -\gamma_{xz}^{a} & \gamma_{xy}^{a}\\ \gamma_{yz}^{a} & 1 & -\gamma_{yx}^{a}\\ -\gamma_{zy}^{a} & \gamma_{zx}^{a} & 1 \end{array}\right]$$

According to the input-output relationship expressed by Equations (22) and (23), the angular velocity and specific force measurement results can be obtained from the pulse output of the IMU.

(29) $$\begin{array}{c} \omega_{ib}^{b}={\left[\begin{array}{ccc} x^{g}\cdot x^{b} & x^{g}\cdot y^{b} & x^{g}\cdot z^{b}\\ y^{g}\cdot x^{b} & y^{g}\cdot y^{b} & y^{g}\cdot z^{b}\\ z^{g}\cdot x^{b} & z^{g}\cdot y^{b} & z^{g}\cdot z^{b} \end{array}\right]}^{-1}{\left[\begin{array}{ccc} S_{x}^{g} & 0 & 0\\ 0 & S_{y}^{g} & 0\\ 0 & 0 & S_{z}^{g} \end{array}\right]}^{-1}\left[\begin{array}{c} N_{x}^{g}-b_{x}^{g}-n_{x}^{g}\\ N_{y}^{g}-b_{y}^{g}-n_{y}^{g}\\ N_{z}^{g}-b_{z}^{g}-n_{z}^{g} \end{array}\right]\\ =K^{g}N^{g}-\omega_{0}-\delta_{\omega } \end{array}$$

(30) $$\begin{array}{c} f^{b}={\left[\begin{array}{ccc} x^{a}\cdot x^{b} & x^{a}\cdot y^{b} & x^{a}\cdot z^{b}\\ y^{a}\cdot x^{b} & y^{a}\cdot y^{b} & y^{a}\cdot z^{b}\\ z^{a}\cdot x^{b} & z^{a}\cdot y^{b} & z^{a}\cdot z^{b} \end{array}\right]}^{-1}{\left[\begin{array}{ccc} S_{x}^{a} & 0 & 0\\ 0 & S_{y}^{a} & 0\\ 0 & 0 & S_{z}^{a} \end{array}\right]}^{-1}\left[\begin{array}{c} N_{x}^{a}-b_{x}^{a}+f_{rxm}^{b}-n_{x}^{a}\\ N_{y}^{a}-b_{y}^{a}+f_{rym}^{b}-n_{y}^{a}\\ N_{z}^{a}-b_{z}^{a}+f_{r=m}^{b}-n_{z}^{a} \end{array}\right]\\ =K^{a}N^{a}-f_{0}+f_{r}^{b}-\delta_{f} \end{array}$$

$K^{g}$ and $K^{a}$ include the scale factors and installation relationship items of the MDRLGs and the accelerometers, respectively, which can be written as:

(31) $$K^{g}={\left[\begin{array}{ccc} S_{x}^{g}\left(x^{g}\cdot x^{b}\right) & S_{x}^{g}\left(x^{g}\cdot y^{b}\right) & S_{x}^{g}\left(x^{g}\cdot z^{b}\right)\\ S_{y}^{g}\left(y^{g}\cdot x^{b}\right) & S_{y}^{g}\left(y^{g}\cdot y^{b}\right) & S_{y}^{g}\left(y^{g}\cdot z^{b}\right)\\ S_{z}^{g}\left(z^{g}\cdot x^{b}\right) & S_{z}^{g}\left(z^{g}\cdot y^{b}\right) & S_{z}^{g}\left(z^{g}\cdot z^{b}\right) \end{array}\right]}^{-1}$$

(32) $$K^{a}={\left[\begin{array}{ccc} S_{x}^{a}\left(x^{a}\cdot x^{b}\right) & S_{x}^{a}\left(x^{a}\cdot y^{b}\right) & S_{x}^{a}\left(x^{a}\cdot z^{b}\right)\\ S_{y}^{a}\left(y^{a}\cdot x^{b}\right) & S_{y}^{a}\left(y^{a}\cdot y^{b}\right) & S_{y}^{a}\left(y^{a}\cdot z^{b}\right)\\ S_{z}^{a}\left(z^{a}\cdot x^{b}\right) & S_{z}^{a}\left(z^{a}\cdot y^{b}\right) & S_{z}^{a}\left(z^{a}\cdot z^{b}\right) \end{array}\right]}^{-1}$$

Assuming that the installation error angle is a small angle, then $K^{g}$ and $K^{a}$ can be written approximately as

(33) $$K^{g}\approx {\left[\begin{array}{ccc} S_{x}^{g} & 0 & 0\\ S_{y}^{g}\gamma_{yz}^{g} & S_{y}^{g} & 0\\ -S_{z}^{g}\gamma_{zy}^{g} & S_{z}^{g}\gamma_{zx}^{g} & S_{z}^{g} \end{array}\right]}^{-1}K^{a}\approx {\left[\begin{array}{ccc} S_{x}^{a} & -S_{x}^{a}\gamma_{xz}^{a} & S_{x}^{a}\gamma_{xy}^{a}\\ S_{y}^{a}\gamma_{yz}^{a} & S_{y}^{a} & -S_{y}^{a}\gamma_{yx}^{a}\\ -S_{z}^{a}\gamma_{zy}^{a} & S_{z}^{a}\gamma_{zx}^{a} & S_{z}^{a} \end{array}\right]}^{-1}$$

Equations (29) and (30) are the calibration parameter models of IMU used in this article, matrix $K^{g}$, $K^{a}$ and zero bias vector $\omega_{0}$,$f_{0}$ are the calibration parameters to be estimated.

#### 3.4.2. Output Error Model of the MDRLG

According to the calibration parameter models of IMU (Equations (29) and (30)), the establishment of the error model of the MDRLG needs to consider the scale factor error, installation error and zero bias. The output error model of MDRLG is as follows:

(34) $$\delta \omega_{ib}^{b}=\left[\begin{array}{ccc} \delta S_{x}^{g} & 0 & 0\\ 0 & \delta S_{y}^{g} & 0\\ 0 & 0 & \delta S_{z}^{g} \end{array}\right]\left[\begin{array}{ccc} 1 & 0 & 0\\ \gamma_{yz}^{g} & 1 & 0\\ -\gamma_{yz}^{g} & \gamma_{yz}^{g} & 1 \end{array}\right]\left[\begin{array}{c} \omega_{ibx}^{b}\\ \omega_{iby}^{b}\\ \omega_{ibz}^{b} \end{array}\right]+\left[\begin{array}{c} \varepsilon_{x}\\ \varepsilon_{y}\\ \varepsilon_{z} \end{array}\right]$$

In Equation (34), $\delta \omega_{ib}^{b}$ is the angular velocity measurement error, $\omega_{ib}^{b}=\left[\begin{array}{ccc} \omega_{ibx}^{b} & \omega_{iby}^{b} & \omega_{ibz}^{b} \end{array}\right]$ is the angular velocity vector in the *b* frame, and $\varepsilon_{I}$ is the zero bias of the MDRLG.

#### 3.4.3. Output Error Model of the Accelerometer

According to the calibration parameter model of IMU, the establishment of the error model of the accelerometer needs to consider the scale factor error, installation error, and zero bias. From Equation (11), it can be seen that the dithering of the MDRLG will generate the size effect error, which needs to be considered in the error model. The output of the accelerometer is sensitive to the working temperature. In this article, the scale factor error caused by temperature change is introduced in the output error model of the accelerometer. The output error model of the accelerometer can be written as follows:

(35) $$\begin{array}{c} \delta f^{b}=\left[\begin{array}{ccc} \delta S_{x}^{a} & 0 & 0\\ 0 & \delta S_{x}^{a} & 0\\ 0 & 0 & \delta S_{x}^{a} \end{array}\right]\left[\begin{array}{ccc} 1 & -\gamma_{xz}^{g} & -\gamma_{xy}^{g}\\ \gamma_{yz}^{g} & 1 & -\gamma_{yx}^{g}\\ -\gamma_{zy}^{g} & \gamma_{zx}^{g} & 1 \end{array}\right]\left[\begin{array}{c} f_{x}^{b}\\ f_{y}^{b}\\ f_{z}^{b} \end{array}\right]+\left[\begin{array}{c} \nabla_{x}\\ \nabla_{y}\\ \nabla_{z} \end{array}\right]+\left[\begin{array}{c} \delta f_{rx}^{b}\\ \delta f_{ry}^{b}\\ \delta f_{r=}^{b} \end{array}\right]\\ +\left[\begin{array}{ccc} \Delta T_{ax}KT_{x} & 0 & 0\\ 0 & \Delta T_{ay}KT_{y} & 0\\ 0 & 0 & \Delta T_{az}KT_{z} \end{array}\right]\left[\begin{array}{c} f_{x}^{b}\\ f_{y}^{b}\\ f_{z}^{b} \end{array}\right] \end{array}$$

In Equation (35), $f^{b}=\left[\begin{array}{ccc} f_{x}^{b} & f_{y}^{b} & f_{z}^{b} \end{array}\right]$ is the specific force vector in the *b* frame, $\nabla_{I}\left(I=x,y,z\right)$ is the zero bias of the MDRLG, $KT_{I}$ is the temperature scale factor error of the MDRLG, $\Delta T_{aI}$ is the temperature change rate, $\delta f_{r}^{b}$ is the size effect error of accelerometers caused by the dithering of the MDRLG, and the Equation is shown in the Equation (10).

## 4. Design of Systematic Calibration Based on Kalman Filter

### 4.1. Outer Lever Arm Effect

When the actual SINS is calibrated, the rotation center of the turntable does not coincide with the sensitive center of the IMU, there is a lever arm vector between the observation point and the sensitive center of the IMU. Assuming that the lever arm vector from the sensitive center of the IMU to the observation point is $l^{b}$. The observation of velocity and position can bewritten as

(36) $$v_{obv}=v_{e}^{n}+C_{b}^{n}\left(\omega_{eb}^{b}\times l^{b}\right)$$

(37) $$p_{obv}=p+\mathrm{diag}\left[\begin{array}{ccc} \frac{1}{R_{M}+h} & 0 & 0\\ 0 & \frac{1}{\left(R_{N}+h\right)\cos L} & 0\\ 0 & 0 & 1 \end{array}\right]C_{b}^{n}l^{b}$$

In Equation (36), $\omega_{eb}^{b}$ is the expression of the rotation angular velocity of the body coordinate frame relative to the earth coordinate frame in the body coordinate frame.

### 4.2. Calibration Filter Design for SINS

In this article, we design a 43-D Kalman filter to estimate all SINS errors during the systematic calibration process. These errors are including general IMU errors, time delay error of accelerometer, temperature error coefficient, inner lever arm error, and outer lever arm error. The state variables of the proposed filter are written as follows:

(38) $$X={\left[\phi^{T}\delta v_{e}^{nT}\delta p^{T}X_{g}^{T}X_{a}^{T}\delta l^{bT}\delta r^{bT}\delta t_{a}\right]}^{T}$$

In Equation (38), $\delta p={\left[\begin{array}{ccc} \delta L & \delta \lambda & \delta h \end{array}\right]}^{T}$ is the position error vector, $X_{g}$ is the error vector of MDRLGs, $X_{a}$ is the error vector of accelerometers, $\delta l^{bT}$ is the outer lever arm vector, $\delta r^{bT}$ is the inner lever arm vector. Based on Equation (34) and Equation (35), $X_{g}$ and $X_{a}$ can be written as follows:

(39) $$\begin{array}{c} X_{a}=\left[\begin{array}{ccccccccc} \delta k_{11}^{a} & \delta k_{21}^{g} & \delta k_{31}^{g} & \delta k_{12}^{g} & \delta k_{22}^{g} & \delta k_{32}^{g} & \delta k_{13}^{a} & \delta k_{23}^{a} & \delta k_{33}^{a} \end{array}\right.\\ \left.\begin{array}{cccccc} \nabla_{x} & \nabla_{y} & \nabla_{z} & \delta KT_{x}^{a} & \delta KT_{y}^{a} & \delta KT_{z}^{a} \end{array}\right] \end{array}$$

Define the navigation coordinate frame as the local geographic coordinate frame (North-Sky-East Coordinate System). The navigation error equation of the inertial navigation frame can be written as follows:

(40) $$\begin{array}{c} \dot{\varphi }=-\omega_{in}^{n}\times \varphi +\delta \omega_{in}^{n}-C_{b}^{n}\delta \omega {{}_{ib}^{b}}^{}\\ \delta v_{e}^{n}=\left(C_{b}^{n}f^{b}\right)\times \varphi -\left(2\omega_{ie}^{n}+\omega_{en}^{n}\right)\times \delta v_{e}^{n}-\left(2\delta \omega_{ie}^{n}+\delta \omega_{en}^{n}\right)\times v_{e}^{n}+\delta g_{l}^{n}+C_{b}^{n}\delta f^{b}\\ \delta \dot{L}=\frac{\delta v_{N}}{R_{M}+h}-\frac{v_{N}\delta h}{{\left(R_{M}+h\right)}^{2}}\\ \delta \dot{\lambda }=\frac{\delta v_{E}}{\left(R_{N}+h\right)\cos L}+\frac{v_{E}\sin L\delta L}{\left(R_{N}+h\right)\cos^{2}L}-\frac{v_{E}\delta h}{{\left(R_{N}+h\right)}^{2}\cos L}\\ \delta \dot{h}=\delta v_{U} \end{array}$$

In Equation (40), φ is the attitude errors, $\omega_{in}^{n}=\omega_{ie}^{n}+\omega_{en}^{n}$ is the projection of the rotation angular velocity of the navigation coordinate frame relative to the inertial coordinate frame in the navigation coordinate frame, $v_{e}^{n}={\left[\begin{array}{ccc} v_{N} & v_{U} & v_{E} \end{array}\right]}^{T}$ is the ground speed, *L*, λ, *h* are the local geographic latitude, longitude and altitude, respectively, $R_{M}$, $R_{N}$ are the radius of the earth’s meridian circle and the radius of the unitary circle, respectively, $\delta \omega_{ib}^{b}$, $\delta f^{b}$ are the angle error and the specific force measurement error, respectively.

The error propagation Equation (40) is rewritten into a matrix form to obtain the state equation of the Kalman filter as follows:

(41) $$\dot{X}=FX+Wu$$

Utilizing Equations (38)–(40), we can obtain the elements of the F matrix as follows:

(42) $$F=\left[\begin{array}{ccccccccc} -\left[\omega_{in}^{n}\times \right] & F_{12} & F_{13} & F_{14} & 0_{3\times 15} & 0_{3\times 3} & 0_{3\times 3} & 0_{3\times 1} & 0_{3\times 1}\\ \left[\left(C_{b}^{n}f^{b}\right)\times \right] & F_{22} & F_{23} & 0_{3\times 9} & F_{25} & 0_{3\times 3} & F_{27} & F_{28} & F_{29}\\ 0_{3\times 3} & F_{32} & F_{33} & 0_{3\times 9} & 0_{3\times 15} & 0_{3\times 3} & 0_{3\times 3} & 0_{3\times 1} & 0_{3\times 3}\\ 0_{9\times 3} & 0_{9\times 3} & 0_{9\times 3} & 0_{9\times 9} & 0_{9\times 15} & 0_{9\times 3} & 0_{9\times 3} & 0_{9\times 1} & 0_{3\times 3}\\ 0_{15\times 3} & 0_{15\times 3} & 0_{15\times 3} & 0_{15\times 9} & 0_{15\times 15} & 0_{15\times 3} & 0_{15\times 3} & 0_{15\times 1} & 0_{3\times 3}\\ 0_{3\times 3} & 0_{3\times 3} & 0_{3\times 3} & 0_{3\times 9} & 0_{3\times 15} & 0_{3\times 3} & 0_{3\times 3} & 0_{3\times 1} & 0_{3\times 3}\\ 0_{3\times 3} & 0_{3\times 3} & 0_{3\times 3} & 0_{3\times 9} & 0_{3\times 15} & 0_{3\times 3} & 0_{3\times 3} & 0_{3\times 1} & 0_{3\times 3}\\ 0_{1\times 3} & 0_{1\times 3} & 0_{1\times 3} & 0_{1\times 9} & 0_{1\times 15} & 0_{1\times 3} & 0_{1\times 3} & 0_{1\times 1} & 0_{3\times 3} \end{array}\right]$$

In Equation (42)

$$F_{12}=\left[\begin{array}{ccc} 0 & 0 & \frac{1}{R_{N}+h}\\ 0 & 0 & \frac{\tan L}{R_{N}+h}\\ \frac{-1}{R_{M}+h} & 0 & 0 \end{array}\right]$$

$$F_{13}=\left[\begin{array}{ccc} -\omega_{ie}\sin L & 0 & \frac{-v_{E}}{{\left(R_{N}+h\right)}^{2}}\\ \omega_{ie}\cos L+\frac{v_{E}}{\left(R_{N}+h\right)\cos^{2}L} & 0 & \frac{-v_{E}\tan L}{{\left(R_{N}+h\right)}^{2}}\\ 0 & 0 & \frac{v_{N}}{{\left(R_{M}+h\right)}^{2}} \end{array}\right]$$

$$F_{14}=-C_{b}^{n}\left[N_{x}^{g}I_{3\times 3}\left[\begin{array}{c} 0_{1\times 2}\\ N_{y}^{g}I_{2\times 2} \end{array}\right]\left[\begin{array}{c} 0_{2\times 1}\\ N_{z}^{g} \end{array}\right]I_{3\times 3}\right]$$

$$F_{22}=-\left[\left(2\omega_{ie}^{n}+\omega_{en}^{n}\right)\times \right]+\left[v_{e}^{n}\times \right]F_{12}$$

$$F_{23}=\left[v_{e}^{n}\times \right]\left[F_{13}+\left[\begin{array}{ccc} -\omega_{ie}\sin L & 0 & 0\\ \omega_{ie}\cos L & 0 & 0\\ 0 & 0 & 0 \end{array}\right]\right]$$

$$F_{25}=C_{b}^{n}\left[\begin{array}{ccccc} N_{x}^{a}I_{3\times 3} & N_{y}^{a}I_{3\times 3} & N_{z}^{a}I_{3\times 3} & I_{3\times 3} & {\left(N^{a}\right)}^{2} \end{array}\right]$$

$$N^{a}=\left[\begin{array}{ccc} N_{x}^{a} & 0 & 0\\ 0 & N_{y}^{a} & 0\\ 0 & 0 & N_{z}^{a} \end{array}\right]$$

$$F_{32}=\left[\begin{array}{ccc} \frac{1}{R_{M}+h} & 0 & 0\\ 0 & 0 & \frac{1}{\left(R_{M}+h\right)}\\ 0 & 1 & 0 \end{array}\right]$$

(43) $$F_{33}=\left[\begin{array}{ccc} 0 & 0 & \frac{-v_{N}}{{\left(R_{M}+h\right)}^{2}}\\ \frac{v_{E}}{\left(R_{N}+h\right)\sin L} & 0 & \frac{-v_{E}}{{\left(R_{N}+h\right)}^{2}\cos L}\\ 0 & 0 & 0 \end{array}\right]$$

Utilizing Equation (12), we can obtain the $F_{27}$ as

(44) $$F_{27}=M_{b}^{a}\cdot \left[{\left(\omega_{ib}^{b}\times \right)}^{2}+\left({\dot{\omega }}_{ib}^{b}\times \right)\right]-\left[\begin{array}{c} 0.5r_{xx}^{b}\left(A_{y}^{2}+A_{z}^{2}\right)\\ 0.5r_{yy}^{b}\left(A_{x}^{2}+A_{z}^{2}\right)\\ 0.5r_{zz}^{b}\left(A_{x}^{2}+A_{y}^{2}\right) \end{array}\right]$$

Utilizing Equation (19), we can obtain $F_{28}$ as:

(45) $$F_{28}=C_{b}^{n}\omega_{ib}^{b}\times f_{SF}^{b}$$

Utilizing Equation (38), we can obtain $F_{29}$ as:

(46) $$F_{29}=\left[\begin{array}{ccc} N_{x}^{a}\Delta T_{ax} & 0 & 0\\ 0 & N_{y}^{a}\Delta T_{ay} & 0\\ 0 & 0 & N_{z}^{a}\Delta T_{az} \end{array}\right]$$

The outer lever arm effect is mainly used in the measurement equation. In order to obtain the velocity and position reference in the Kalman filter, we take the outer lever arm effect into consideration. The measurement variables function is written as:

(47) $$Z=\left[\begin{array}{c} v_{e}^{n}+C_{b}^{n}\left[\left(\omega_{ib}^{b}-C_{b}^{nT}\omega_{ie}^{n}\right)\times \delta l^{b}\right]-v_{obv}\\ p+diag\left(\frac{1}{R_{M}+h},\frac{1}{\left(R_{N}+h\right)\cos L},1\right)C_{b}^{n}\delta l^{b}-p_{obv} \end{array}\right]=HX+V$$

The measurement matrix *H* can be written as:

(48) $$H=\left[\begin{array}{ccccccc} H_{11} & I_{3\times 3} & H_{13} & H_{14} & 0_{3\times 15} & C_{b}^{n}\left[\omega_{eb}^{b}\times \right] & 0_{3\times 7}\\ H_{21} & 0_{3\times 3} & H_{23} & 0_{3\times 9} & 0_{3\times 15} & H_{26} & 0_{3\times 7} \end{array}\right]$$

Utilizing Equations (39)–(41), we can obtain the elements of *H* as:

$$H_{11}=\left[\left(C_{b}^{n}\left(\omega_{eb}^{b}\times l^{b}\right)\right)\times \right]-C_{b}^{n}\left[l^{b}\times \right]C_{n}^{b}\left[\omega_{ie}^{n}\times \right]$$

$$H_{13}=C_{b}^{n}\left[l^{b}\times \right]C_{n}^{b}\left[\begin{array}{ccc} -\omega_{ie}\sin L & 0 & 0\\ \omega_{ie}\cos L & 0 & 0\\ 0 & 0 & 0 \end{array}\right]$$

$$H_{14}=-C_{b}^{n}\left[l^{b}\times \right]\left[N_{x}^{g}I_{3\times 3}\left[\begin{array}{c} 0_{1\times 2}\\ N_{y}^{g}I_{2\times 2} \end{array}\right]\left[\begin{array}{c} 0_{2\times 1}\\ N_{z}^{g} \end{array}\right]I_{3\times 3}\right]$$

$$H_{23}=\left[\begin{array}{ccc} 1 & 0 & \frac{-l_{x}^{n}}{{\left(R_{M}+h\right)}^{2}}\\ \frac{l_{y}^{n}\sin L}{\left(R_{N}+h\right)\cos^{2}L} & 1 & \frac{-l_{y}^{n}}{{\left(R_{N}+h\right)}^{2}\cos L}\\ 0 & 0 & 1 \end{array}\right]$$

(49) $$H_{26}=diag\left[\frac{1}{R_{M}+h},\frac{1}{\left(R_{N}+h\right)\cos L},1\right]$$

The state model and the measurement model are both linear, and we utilize the Kalman filter to estimate the state variables. Kalman filtering process is organized as follows:

(1) One step prediction of state

(50) $${\hat{X}}_{k,k-1}=\Phi_{k,k-1}{\hat{X}}_{k-1}$$

In Equation (50), ${\hat{X}}_{k-1}$ is the state variable at time k−1, ${\hat{X}}_{k,k-1}$ is the one step prediction of state variable, $\Phi_{k,k-1}$ is the state transition matrix.

(2) One step prediction of mean square error

(51) $$P_{k,k-1}=\Phi_{k,k-1}P_{k-1}\Phi_{k,k-1}^{T}+\Gamma_{k-1}Q_{k-1}\Gamma_{k-1}^{T}$$

In Equation (51), $P_{k-1}$ is the error matrix at time k−1, $P_{k,k-1}$ is the prediction error matrix, $Q_{k-1}$ is the covariance matrix at time k−1,$\Gamma_{k-1}$ is the input control matrix at time k−1.

(3) Gain matrix

(52) $$K_{k}=P_{k,k-1}H_{k}^{T}{\left(H_{k}P_{k,k-1}H_{k}^{T}+R_{k}\right)}^{-1}$$

In Equation (52), $H_{k}$ is the observation matrix at time *k*, $K_{k}$ is the gain matrix at time *k*, $R_{k}$ is the measurement noise covariance matrix at time *k*.

(4) State estimation

(53) $${\hat{X}}_{k}={\hat{X}}_{k,k-1}+K_{k}\left(Z_{k}-H_{k}{\hat{X}}_{k,k-1}\right)$$

In Equation (53), ${\hat{X}}_{k}$ is the state variable at time *k*, $Z_{k}$ is observation matrix at time *k*.

(5) Mean square error estimation

(54) $$P_{k}=\left(I-K_{k}H_{k}\right)P_{k,k-1}$$

In Equation (54), *I* is the unit matrix.

**Figure 4:**
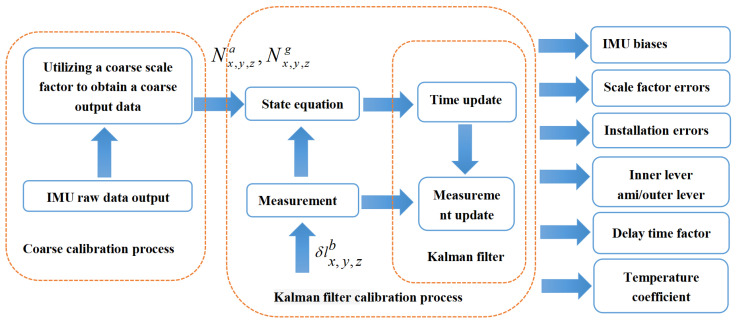
Schematic diagram of size effect.

The systematic calibration process of SINS is shown in Figure 4. The diagram consists of the coarse calibration process and the calibration process that utilizes the Kalman filter. In the first process, we can obtain an inaccurate IMU output $N_{x,y,z}^{g}$ and $N_{x,y,z}^{a}$ to guarantee the linearity of the Kalman filter. In the second process, the state equation is updated by the output $N_{x,y,z}^{g}$ and $N_{x,y,z}^{a}$ of the IMU, and the measurement equation is updated by entering the initial value of the error of outer lever arms $\delta l_{x,y,z}^{b}$. Finally, we use the Kalman filter to estimate SINS error. After the second process, we can obtain the SINS errors that we want.

**Figure 5:**
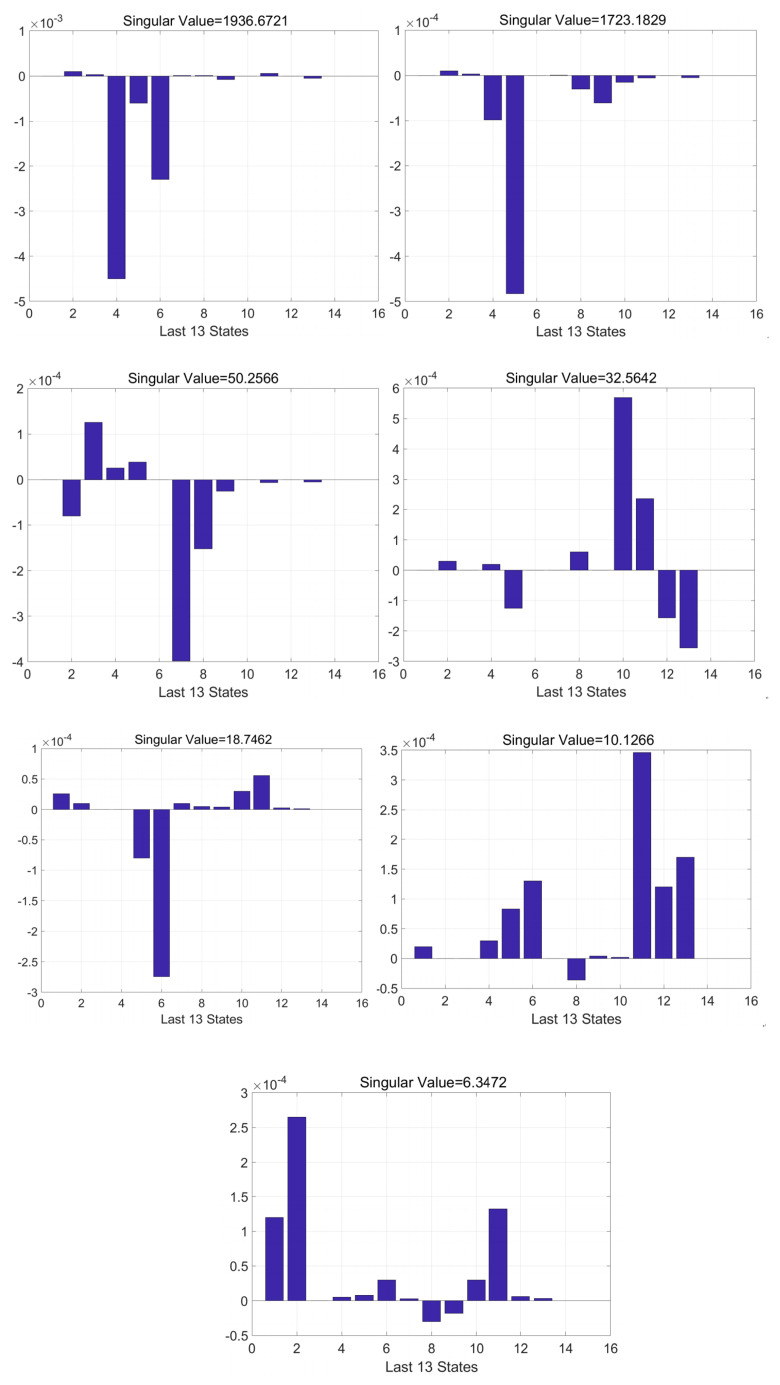
Observability analysis.

The rotation excitation method we adopt is the arrangement method designed by Camberlein [7]. This rotation excitation method can effectively calibrate the zero bias, scale factor error, and installation error of the inertial device. The Euler angle change of IMU in the systematic calibration is shown in Figure 5. The temperature of the SINS rises when it is operating. We use this temperature change as a temperature excitation for systematic calibration. During the systematic calibration test, we pasted the DS18B20 temperature sensor on the surface of the accelerator to collect the temperature of the accelerometer in real-time.

### 4.3. Analysis of Observable Degree

To verify the validity of the 43-dimensional Kalman filter model designed in the previous section to excite the SINS error parameters, an observability matrix (SOM) is introduced to discuss the observability of the system. The observability matrix $Q_{s}\left(r\right)$ is

(55) $$Q_{s}\left(r\right)=\left[\begin{array}{ccccc} Q_{1}^{T} & Q_{2}^{T} & Q_{3}^{T} & L & {\left.Q_{r}^{T}\right]}^{T} \end{array}\right.$$

When the rank of SOM $Q_{s}\left(r\right)=n$, the system is completely observable, if $Q_{s}\left(r\right)<n$, the system is not completely observable. In order to intuitively judge the convergence speed and accuracy of the state variable estimation, SVD analysis is carried out on the basis of PWCS to calculate the observable degree of each state variable. The larger the singular value corresponding to the system state variable, the higher the corresponding observability, and the easier it is to estimate in Kalman filtering. Perform singular value decomposition of SOM:

(56) $$Q_{s}\left(r\right)=U\sum V^{T}$$

In Equation (56): Both matrices *U* and *V* are orthogonal matrices. $\sum ={\left[\begin{array}{cc} S_{rxr} & 0_{rx|m-r|} \end{array}\right]}^{T}$, where *S* is the diagonal matrix, $\mathrm{diag}\left\{\begin{array}{cccc} \sigma_{1} & \sigma_{2} & \cdots & \sigma_{r} \end{array}\right\}$ is called the singular value of matrix $Q_{s}\left(r\right)$. According to the projection transformation relationship, projection of initial vector $X_{0}$ in space extended by $\left[\sigma_{1}v_{1},\sigma_{2}v_{2},\cdots \sigma_{r}v_{r}\right]$ is transformed to observation value *Y*. Therefore, at least *r* observation values are required to uniquely determine the state $X_{0}$. When $\sigma_{1}>0$, the initial state can be determined according to the measurement information:

(57) $$X_{0}=\sum_{i=1}^{r}\left(\frac{u_{i}^{T}Y}{\sigma_{i}}\right)v_{i}$$

According to Equation (56), the singular value of the SOM matrix represents the radius of the ellipsoid formed by the estimated initial state. The larger the singular value, the smaller the radius of the ellipsoid, the stronger the estimation of the initial state and the more observable the state variable is. The expression of observability is as follows:

(58) $$\eta_{j}=\sigma_{i}/\sigma_{0}i=12\ldots$$

In Equation (58): $\eta_{j}$ represents the observability of the *j*-th state component, $\sigma_{0}$ represents the singular value of external observations, and $\sigma_{i}$ is the singular value that maximizes $u_{i}^{T}Yv_{i}/\sigma_{i}\left(i=12\ldots r\right)$.

In this article, we use the above methods to analyze the 43-D Kalman filter system. After the filtering, the rank of the $Q_{s}\left(r\right)$ matrix is 43 (full rank), indicating that all the state variable of the system is observable. The graph in Figure 4 is the observability histogram of the last 13 state variable. The order of the abscissa is $\delta KT_{x}^{a},\delta KT_{y}^{a},\delta KT_{z}^{a},\delta r_{x}^{b},\delta r_{y}^{b},\delta r_{z}^{b},\delta l_{x}^{b},\delta l_{y}^{b}$, $\delta l_{z}^{b},\delta t_{a},\delta KT_{x}^{a},\delta KT_{y}^{a},\delta KT_{z}^{a}$. There are seven state variable ($\delta r_{x}^{b},\delta r_{y}^{b},\delta r_{z}^{b},\delta t_{a},\delta KT_{x}^{a},\delta KT_{y}^{a},\delta KT_{z}^{a}$) among them are the newly introduced component in this article. Their observability is shown in Figure 5.

The larger the SV value of the corresponding state variable, the better the observability of the corresponding state. If the diagonal element is greater than 10${}^{-4}$, it is considered to be a non-zero element, then the corresponding state variable is observable. Figure 5 shows the observed histogram of each state variable over five time periods during the calibration process. We can see that each state variable has a SV value greater than 10${}^{-4}$ that occurs at least once in five observation degree histograms, indicating that each state variable is significant. Each error parameter that needs to be calibrated can be effectively stimulated, so the calibration method is effective.

## 5. Test Results and Analysis

### 5.1. Calibration Test

**Figure 6:**
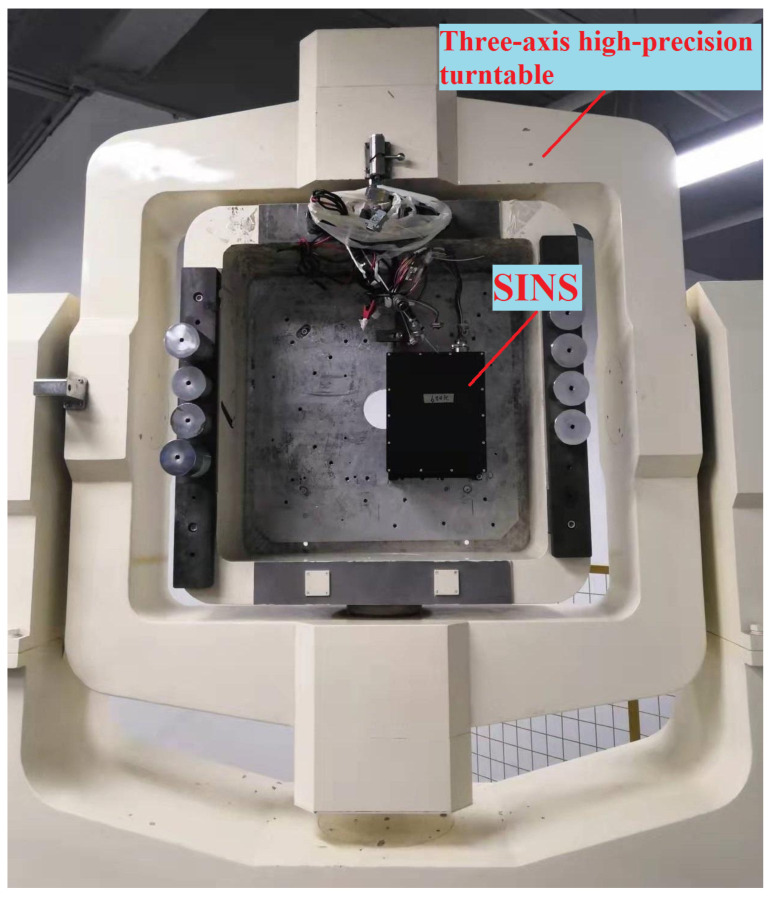
The diagram of the experimental system.

The calibration experimental system includes a high precision three-axis turntable, a certain type of SINS with MDRLG, turntable control terminal, and SINS data acquisition device. The angular resolution of the three-axis turntable used in the experiment is better than 1 arc second. The IMU of the SINS used in the calibration experiment is equipped with three MDRLGs and three accelerometers. The bias stability of the MDRLG and the accelerometers are $0.005^{\circ }$/h and 35μg, respectively. A DS18B20 temperature sensor made by Maxim (an American semiconductor company) is installed on the surface of each accelerometer to measure the surface temperature of the accelerometer. The test equipment is shown in Figure 6.

**Figure 7:**
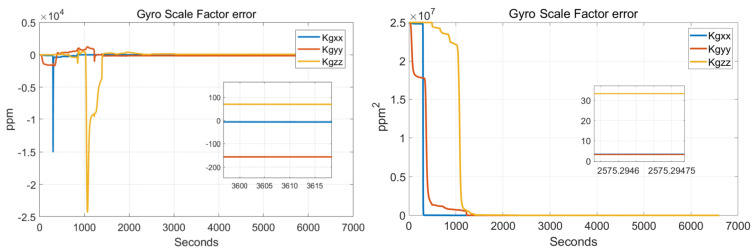
Gyro-scale factor errors.

**Figure 8:**
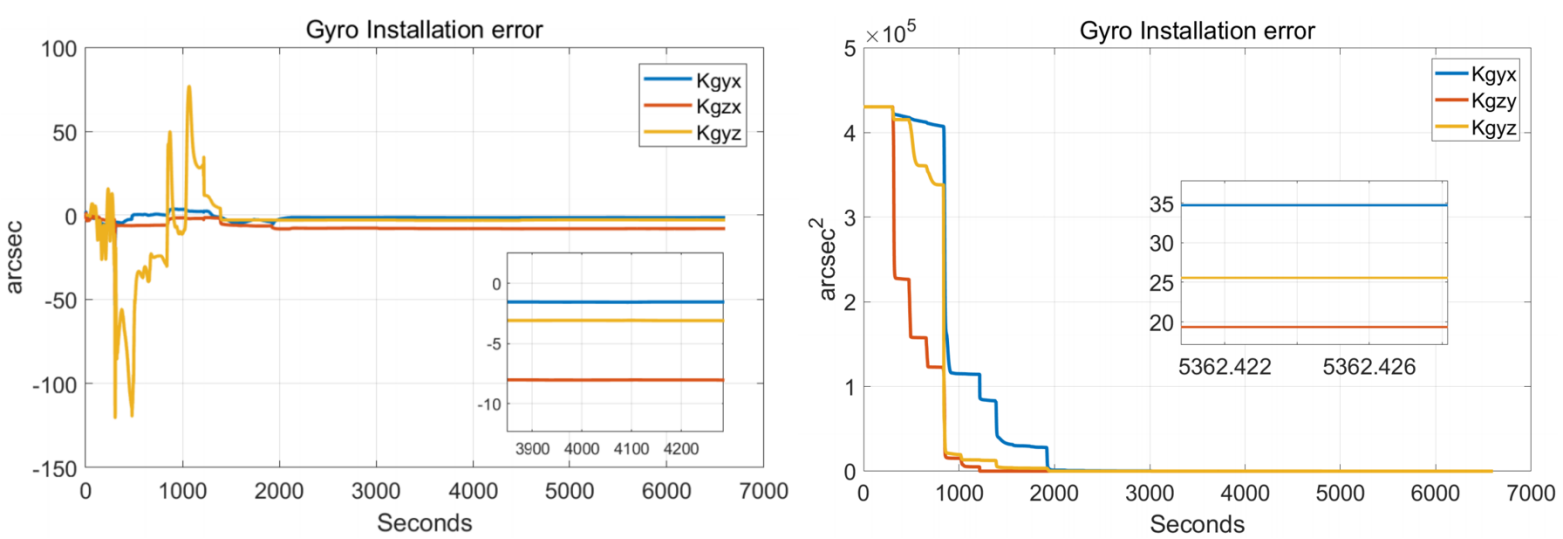
Gyro installation errors.

**Figure 9:**
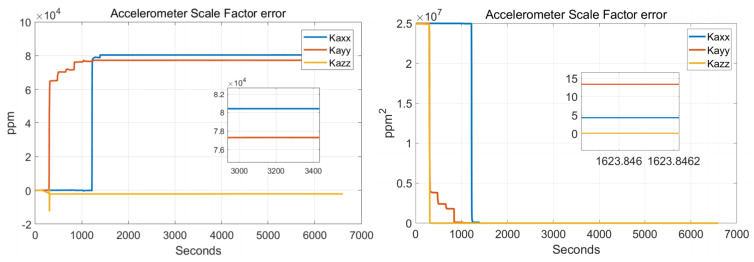
Accelerometer scale factor errors.

**Figure 10:**
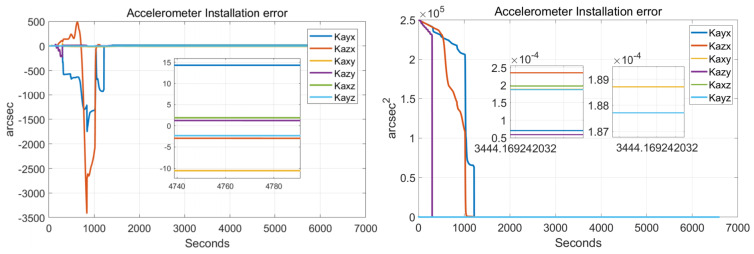
Accelerometer installation errors.

**Figure 11:**
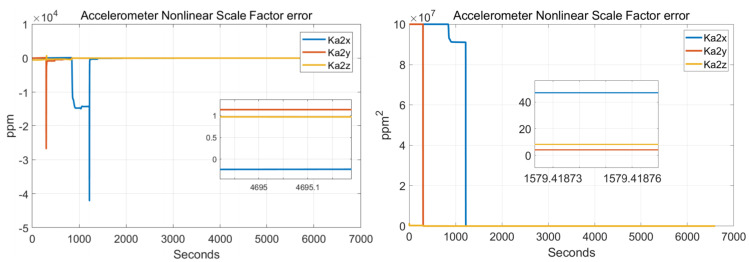
Accelerometer nonlinear scale factor errors.

**Figure 12:**
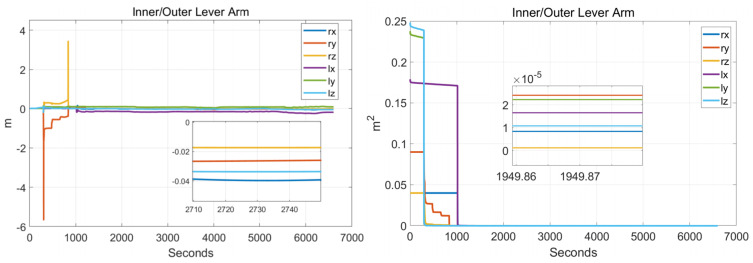
Inner/outer lever arms.

**Figure 13:**
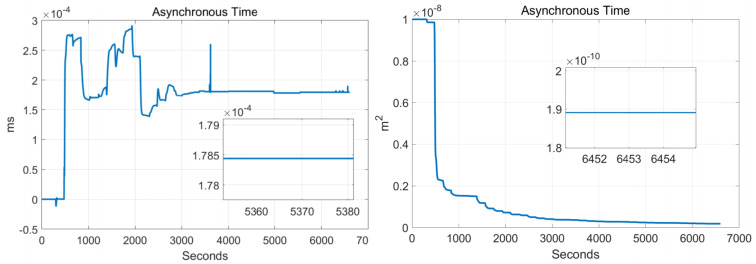
Asynchronous time.

**Figure 14:**
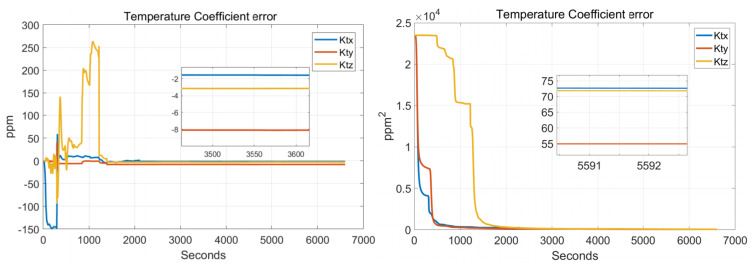
Temperature coefficient errors.

In order to verify the effectiveness of the systematic calibration results, we need to analyze the variable of the Kalman filter during the process of actual systematic calibration. In order to judge whether the state variables of the Kalman filter converges, the estimate covariance (diagonals) has to be drawn. If the state variable is observable, the diagonal element of the corresponding estimate covariance matrix (P matrix) needs to converge to zero. The state estimation curve has to converge to a constant. The estimation curves during the systematic calibration process are shown in Figure 7, Figure 8, Figure 9, Figure 10, Figure 11, Figure 12, Figure 13 and Figure 14.

The diagonal elements of the P matrix are starting to converge after about 2000 s, and the convergence time of the state estimation curve is consistent with the estimation covariance curve. Combined with the previous SVD-based observability analysis, we can conclude that the 43-D Kalman filter is observable when utilizing the rotation path shown in Table 1. All IMU error parameters are starting to converge after about 2000 s, indicating that the calibration method proposed in this article can effectively calibrate the temperature error coefficient of accelerometer, time delay coefficient of accelerometer, and inner lever arm parameters. At the same time, it can effectively calibrate conventional IMU error parameters such as IMU scale factor error, installation error, and zero bias.

### 5.2. Static Swing Navigation Test

To verify the error feedback effect of the error parameters, a three-axis turntable swing navigation experiment is carried out. The rocking motion can fully stimulate the lever arm effect, which is used to verify the feedback effect of the calibration parameters. The swing mode of the turntable is shown in Table 2. The total duration of the navigation experiment is 48 h.

To verify the error compensation effects of mechanical dithering of the MDRLG, the temperature error coefficient, the time delay coefficient of the accelerometer and inner lever arm in the navigation test. Under the same 18-sequence calibration path, the filters shown in Table 1 are used for system-level calibration. Each filter contains error items are shown in Table 3.

**Figure 15:**
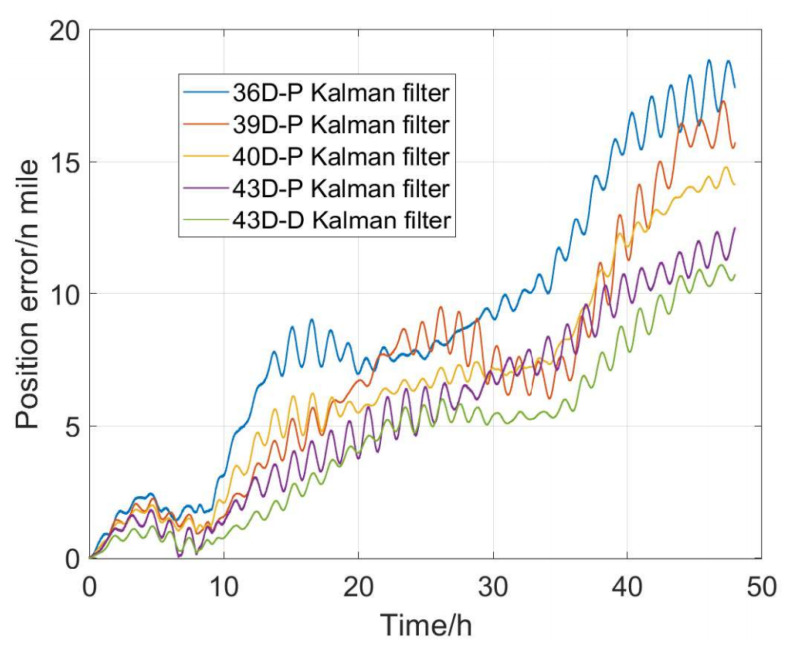
The positioning errors of navigation.

In Table 3, the filter model of type P is the calibration model that does not consider the mechanical dithering of the MDRLG, and the filter model of type B is the calibration model that takes the mechanical dithering of the MDRLG into consideration. We use the calibration results of each filter model to perform the navigation solution experiment at the same time, and the horizontal positioning error of the navigation experiment is shown in Figure 15. The horizontal position accuracy from high to low is the 43D-B filter, 43D-P filter, 40D-P filter, 39D-P filter, 36D-P filter, indicating that the calibration of each parameter is effective. Comparing the navigation accuracy based on 40D-P filter compensation with that based on 43D-P filter compensation, it can be concluded that the navigation accuracy of the SINS after temperature compensation of accelerometer is significantly improved. The maximum horizontal positioning error of navigation based on 36D-P filter calibration exceeds18.7 nautical miles. The accuracy of each stage of 48-h navigation based on 43D-B dimensional filter calibration is significantly better than other filters. The maximum horizontal positioning error is better than 11.2 nautical miles, and navigation accuracy increased by 40.1%. It can be seen that using the systematic calibration method proposed in this article to feedback the IMU calibration parameters can effectively improve the navigation accuracy of the SINS.

## 6. Conclusions

Aiming at the problem of high-precision calibration of SINS, in this article we propose a systematic calibration method based on a 43-D Kalman filter. Algorithm analysis and experimental results show that:

(1) The size effect error of dithering of the MDRLG compensation method derived in this article can effectively improve navigation accuracy. The analysis shows that the influence of dithering of the MDRLG on the navigation accuracy is mainly related to the amplitude of dithering, and the relationship with the frequency of dithering is not obvious. The velocity error and position error caused by the dithering of the MDRLG are, respectively, a linear function and a quadratic function of time.

(2) An error model of the accelerometer delay time and specific force measurement is established. The velocity error and position error caused by the time delay of the accelerometer are a linear function and a quadratic function of time, respectively.

(3) In order to solve the above-mentioned problem, we derive a continuous linear 43-D SINS error model considering the above-mentioned two error parameters and expand the temperature coefficients of accelerometers, inner lever arm error, outer lever arm error parameters to achieve high-precision calibration of SINS. Observability analysis shows that after the 18-bit rotation sequence, each state is observable. The calibration test shows that the calibration method can effectively calibrate all state errors. The static swing test shows that after the calibration parameters are compensated for multiple error sources, the maximum positioning error of the 48-hour navigation level is 11.2 nautical miles. Compared with the calibration that does not consider the compensation for dithering of the MDRLG, the time delay of accelerometer, temperature coefficient of accelerometer, and inner lever arm error, navigation accuracy has been improved by 40.1%.

## Figures and Tables

**Table 1:** Rotation path of systematic calibration.

Number	Rotation Angle/Axis	Attitude after Rotation (XYZ)
1	+90Y	NED
2	+180Y	UEN
3	+180Y	DES
4	+90Z	UEN
5	+180Z	EDN
6	+180Z	WUN
7	+90X	EDN
8	+180X	ENU
9	+180X	ESD
10	+90X	ENU
11	+90X	EUS
12	+90X	ESD
13	+90Z	EDN
14	+90Z	DWN
15	+90Z	WUN
16	+90Y	UEN
17	+90Y	SEU
18	+90Y	DES

**Table 2:** Vibration patterns.

Vibration Axis (IMU)	Amplitude	Frequency
*x*-axis	2°	0.4
*y*-axis	3°	0.3
*z*-axis	4°	0.4

**Table 3:** Each Kalman filter model contains error components.

Filter Model	Contains Error Components
36D-P Kalman filter	IMU scale factor error, installation error, zero offset, outer lever arm error
39D-P Kalman filter	IMU scale factor error, installation error, zero offset, outer lever arm error
	inner lever arm error $\delta r^{bT}$
40D-P Kalman filter	IMU scale factor error, installation error, zero offset, outer lever arm error
	inner lever arm error $\delta r^{bT}$, time delay factor $\delta t_{a}$
43D-P Kalman filter	IMU scale factor error, installation error, zero offset, outer lever arm error
	inner lever arm error $\delta r^{bT}$, time delay factor $\delta t_{a}$
	Temperature error coefficient $k_{tI}\left(I=x,y,z\right)$
43D-B Kalman filter	IMU scale factor error, installation error, zero offset, outer lever arm error
(Consider the dithering	inner lever arm error $\delta r^{bT}$, time delay factor $\delta t_{a}$
of MDRLG	Temperature error coefficient $k_{tI}\left(I=x,y,z\right)$
compensation model)	

## Data Availability

Not applicable.

## Sample Availability

Samples of the compounds are available from the authors.

## References

[1] Ren Q., Wang B., Deng Z., Fu M. (2014). Amulti-position self-calibration method for dual-axis rotational inertial navigation system. Sens. Actuators A Phys..

[2] Nieminen T., Kangas J., Suuriniemi S., Kettunen L. (2010). An enhanced multi-position calibration method for consumer-grade inertial measurement units applied and tested. Meas. Sci. Technol..

[3] Zhang H., Wu Y., Wu W., Wu M., Hu X. (2010). Improved multi-position calibration for inertial measurement units. Meas. Sci. Technol..

[4] Pittman D.N., Roberts C.E. Determining Inertial Errors from Navigation-in-Place Data. Proceedings of the IEEE Position Location and Navigation Symposium.

[5] Savage P.G. (2007). Strapdown Analytics.

[6] Zhou Z.H., Qiu H.B., Li Y., Lian T., Wang T. (2010). Systematic calibration method for SINS with low-precision two-axis turntable. J. Chin. Inert. Technol..

[7] Camberlein L., Mazzanti F. Calibration technique for laser gyro strapdown inertial navigation systems. Proceedings of the Symposium Gyro Technology.

[8] Joos D.K., Hunsanger W. High Accuracy Laboratory Tests on an Orbital-Microgravity-Sensor-System. Proceedings of the Symposium Gyro Technology.

[9] Grewal M.S., Henderson V.D., Miyasako R.S. (1991). Application of Kalman Filtering to the Calibration and Alignment of Inertial Navigation Systems. IEEE Trans. Autom. Control..

[10] Cai Q., Yang G., Song N., Liu Y. (2016). Systematic Calibration for Ultra-High Accuracy Inertial Measurement Units. Sensors.

[11] Yu X.D., Wang Y., Zhang P.F., Xie Y.P., Tang J.X., Long X.W. (2012). Calibration of RLG drift in single-axis rotation INS. Opt. Precis. Eng..

[12] Liu B., Ren J., Bai H. (2017). Systematic Calibration Method Based on High-order Kalman Filter for Laser Gyro SINS. Missilesand Space Veh..

[13] Shi W., Wang X., Zheng J., Wang Y. (2016). Multi-position systematic calibration method for RLG-SINS. Infrared Laser Eng..

[14] Yu H. (2012). Research on the Methods for Improving the Accuracy of Laser Gyro SINS in Vibration Environment. Ph.D. Thesis.

[15] Weng J. (2020). Multi-position continuous rotate-stop fast temperature parameters estimation method of flexible pendulum accelerometer triads. Measurement.

[16] Gao P., Li K., Song T., Liu Z. (2017). An accelerometers-size-effect self-calibration method for triaxis rotational inertial navigation system. IEEE Trans. Ind. Electron..

[17] Song T., Li K., Wu Q., Li Q., Xue Q. (2020). An improved self-calibration method with consideration of inner lever- arm effect for dual-axis RINS. Meas. Sci. Technol..

[18] Xu C., Miao L., Zhou Z. (2019). A self-calibration method of inner lever arms for dual-axis rotation INS. Meas. Sci. Technol..

[19] Jiang Q., Tang J., Han S., Bao Y. (2015). Systematic calibration method based on 36-dimension Kalman filter for laser gyro SINS. Infrared Laser Eng..

[20] Gao J.M., Zhang K.B., Chen F.B., Yang H.B. (2015). Temperature characteristics and error compensation for quartz flexible accelerometer. Int. J. Autom. Comput..

[21] Pan Y., Li L., Ren C., Luo H. (2010). Study on the compensation for a quartz accelerometer based on a wavelet neural network. Meas. Sci. Technol..

[22] Ban J., Wang L., Liu Z., Zhang L. (2020). Self-calibration method for temperature errors in multi-axis rotational inertial navigation system. Opt. Express.

